# Identification of *MEN1* gene mutations in families with MEN 1 and related disorders

**DOI:** 10.1054/bjoc.2000.1380

**Published:** 2000-10

**Authors:** L Bergman, B Teh, J Cardinal, J Palmer, M Walters, J Shepherd, D Cameron, N Hayward

**Affiliations:** Queensland Cancer Fund Research Unit, Joint Experimental Oncology Programme of the Queensland Institute of Medical Research and the University of Queensland, Herston, QLD, 4006, Australia; Department of Surgery, University of Tasmania, Hobart, TAS, 7000, Australia; Clinical Genetics Unit, Department of Molecular Medicine, Karolinska Hospital, Sweden; Department of Diabetes and Endocrinology, Princess Alexandra Hospital, Woolloongabba, QLD, 4102, Australia

**Keywords:** MEN 1, mutation analysis, FIHPT

## Abstract

Following identification of the *MEN1* gene, we analysed patients from 12 MEN 1 families, 8 sporadic cases of MEN 1, and 13 patients with MEN 1-like symptoms (e.g. cases of familial isolated hyperparathyroidism (FIHPT), familial acromegaly, or atypical MEN 1 cases) for the presence of germline *MEN1* mutations. The entire coding region of the *MEN1* gene was sequenced, and mutations were detected in 11 MEN 1 families; one sporadic MEN 1 patient, one case of FIHPT and one MEN 1-like case. Constitutional DNA samples from individuals without *MEN1* mutations were digested with several restriction enzymes, Southern blotted and probed with *MEN1* cDNA to analyse for the presence of larger deletions of the *MEN1* gene unable to be detected by PCR. One MEN 1 patient was found to carry such a deletion. This patient was heterozygous for the D418D polymorphism, however sequence analysis of RT-PCR products showed that only the variant allele was transcribed, thus confirming the result obtained by Southern analysis, which indicated loss of a region containing the initiation codon of one allele. © 2000 Cancer Research Campaign


*British Journal of Cancer* (2000) **83**(8), 1009–1014
© 2000 Cancer Research Campaign
doi: 10.1054/ bjoc.2000.1380, available online at http://www.idealibrary.com on IDEAL®

### Identification of *MEN1* gene mutations in families with MEN 1 and related disorders

**L Bergman, B Teh[1,2], J Cardinal[3], J Palmer, M Walters, J Shepherd[1], D Cameron[3] and N Hayward**

Queensland Cancer Fund Research Unit, Joint Experimental Oncology Programme of the Queensland Institute of Medical Research and the University of Queensland, Herston QLD 4006, Australia; [1]Department of Surgery, University of Tasmania, Hobart TAS 7000, Australia; [2]Clinical Genetics Unit, Department of Molecular Medicine, Karolinska Hospital, Sweden; [3]Department of Diabetes and Endocrinology, Princess Alexandra Hospital, Woolloongabba, QLD 4102, Australia

**Summary** Following identification of the *MEN1* gene, we analysed patients from 12 MEN 1 families, 8 sporadic cases of MEN 1, and 13 patients with MEN 1-like symptoms (e.g. cases of familial isolated hyperparathyroidism (FIHPT), familial acromegaly, or atypical MEN 1 cases) for the presence of germline *MEN1* mutations. The entire coding region of the *MEN1* gene was sequenced, and mutations were detected in 11 MEN 1 families; one sporadic MEN 1 patient, one case of FIHPT and one MEN 1-like case. Constitutional DNA samples from individuals without *MEN1* mutations were digested with several restriction enzymes, Southern blotted and probed with *MEN1* cDNA to analyse for the presence of larger deletions of the *MEN1* gene unable to be detected by PCR. One MEN 1 patient was found to carry such a deletion. This patient was heterozygous for the D418D polymorphism, however sequence analysis of RT-PCR products showed that only the variant allele was transcribed, thus confirming the result obtained by Southern analysis, which indicated loss of a region containing the initiation codon of one allele. © 2000 Cancer Research Campaign

**Keywords:** MEN 1; mutation analysis; FIHPT

Multiple endocrine neoplasia type 1 (MEN 1) is an autosomal dominant disorder characterized by an inherited predisposition to neoplasia and hyperplasia of the parathyroid glands, anterior pituitary and endocrine pancreas. Also observed are thymic and lung carcinoids, adrenocortical tumours, lipomas, thyroid tumours (Komminoth et al, 1998) and skin lesions such as angiofibromas and collagenomas (Darling et al, 1997). MEN 1 has an estimated prevalence of 0.02–0.2/1000, depending on race and geographic location (Teh et al, 1995). The disease shows almost complete penetrance by age 50 (Komminoth et al, 1998).

The gene responsible for MEN 1 is located on chromosome 11q13, and was shown by linkage analysis and tumour loss of heterozygosity studies to conform to Knudson's two-hit model of tumorigenesis (Larsson et al, 1998). The *MEN1* tumour suppressor gene was recently identified by positional cloning (Chandrasekharappa et al, 1997). The gene covers approximately 8 kilobases (kb) of genomic DNA, is comprised of ten exons and encodes a 2.8 kb ubiquitously expressed mRNA, producing a putative 610 amino acid protein showing no homology to any others in the current databases.

Numerous studies aimed at identifying predisposing *MEN1* gene mutations in MEN 1 families and patients with related disorders have been reported in the literature (for example Agarwal et al, 1997; Bassett et al, 1998; Giraud et al, 1998; Teh et al 1998a). No apparent genotype–phenotype correlations have been identified. Mutations are evenly distributed throughout the coding region of the gene, without obvious hotspots suggestive of functional domains.

In this study, we aimed to characterize the *MEN1* gene for mutations in 12 Australian MEN 1 kindreds and in 21 cases displaying MEN 1-like symptoms, to add to the extensive list of mutations previously reported, and assist with pre-symptomatic testing and genetic counselling of members of these patients' families.

*Received 18 October 1999*
*Revised 2 May 2000*
*Accepted 15 June 2000*

*Correspondence to:* N Hayward

### SUBJECTS, MATERIALS AND METHODS

### Patients

Patients were primarily obtained from major public teaching hospitals in Brisbane, as well as from endocrinologists throughout Australia who referred them specifically for mutation testing of the *MEN1* gene. A patient was considered to have familial MEN 1 if they had at least one affected first-degree relative, and provided one or more family members presented with at least two of the three major endocrine lesions (parathyroid, pancreas, anterior pituitary). Sporadic MEN 1 was diagnosed if a patient had at least two of the three major MEN 1 lesions, with no known family history of any endocrine manifestation. Patients were classified as MEN 1-like if they did not fit the above definition of MEN 1, but one of the major MEN 1 lesions was present in at least one family member. Diagnosis of familial isolated hyperparathyroidism (FIHPT) was based on the presence of primary hyperparathyroidism in at least one first-degree relative, in the absence of any other endocrine manifestations. Similar criteria were used for diagnosis of familial acromegaly. A detailed clinical and family history was taken from each available family member, and a peripheral blood sample was drawn for DNA analysis, for which informed consent was given.

**Table 1** PCR primers used for mutation analysis

| Exon/Gene segment | Primer sequence (5′–3′) | Annealing temp. (°C) | Annealing position[a] |
|---|---|---|---|
| *MEN1* cDNA | | | |
| 1F | CTAGAGATCCCAGAAGCCAC | 56 | 20–39 |
| R | CACTACCCAGGCATGATCC | | 665–647 |
| 2F | ACAGGCACCAAATTGGACAG | 58 | 552–571 |
| R | CACATTGCGGTTGCGACAG | | 1112–1094 |
| 3F | CTGGCTGCTCTATGACCTG | 58 | 902–920 |
| R | CTAGGGACTGCACAAGAAAG | | 1443–1424 |
| 4F | CGACGGCATCTGCAAATGGG | 60 | 1361–1380 |
| R | GGGTTTGGGTAGAGGTGAGG | | 2059–2040 |
| *MEN1* gene | | | |
| 2F | GTGAGCAGAGGCTGAAGAGG | 64 | 2130–2149 |
| R | ATAACACCTGCCGAACCTCAC | | 2844–2824 |
| 3F | AGGTTGGGTCACAGGCTTG | 58 | 4181–4199 |
| R | CTATGTGGGTGGTGATGGG | | 4617–4599 |

[a] Sequence taken from GenBank accession number U93237 (gDNA) and U93236 (cDNA)

### DNA and RNA isolation

DNA was isolated from whole blood or lymphoblastoid cell lines (LCLs) using the salt extraction method of Miller et al (1986). Total RNA was isolated from LCL pellets using an RNeasy Mini kit (QIAGEN) as per the manufacturer's instructions.

### PCR

Each of the nine coding exons of the *MEN1* gene were amplified using the polymerase chain reaction (PCR). The primers for exons 4–10 were obtained from the European Consortium for MEN 1 and are reported in Lemmens et al (1997). Primers used for amplifying exons 2 and 3 are listed in Table 1. Reactions were performed in 50 μl volumes containing 10 pmol each primer, 5% DMSO, 0.75 U Amplitaq Gold (Perkin Elmer) and approximately 50 ng of template DNA. For PCR of exon 2, 1M betaine was substituted for DMSO. Reactions were cycled on either a Perkin Elmer Cetus or Hybaid Omnigene thermal cycler under the following conditions: 95°C for 12 minutes, followed by 35 cycles of 95°C for 1 minute, 62°C for 1 minute (this annealing temperature differed for some primers; see Table 1), and 72°C for 90 seconds. PCR products were purified by agarose gel electrophoresis and DNA was isolated from the agarose using a QIAquick gel extraction kit (QIAGEN) as per the manufacturer's instructions.

### RT-PCR

Five μg of total RNA were used to synthesize the first strand of cDNA using an oligo$(dT)_{12-18}$ primer (Boehringer Mannheim) and a SuperScript II reverse transcriptase kit (Gibco BRL) as per the manufacturer's instructions. Reverse transcriptase PCR (RT-PCR) was performed using the cDNA primers listed in Table 1. Cycling conditions were the same as those described for genomic PCR, using the annealing temperatures listed in Table 1. PCR products were purified as described for genomic PCR.

### DNA sequencing

Standard protocols were used for cycle sequencing of PCR products using Big Dye dye terminator reaction premix (ABI prism). Primers used were the same as for PCR. Cycling reactions were performed on a Selby TS-MP96 thermal cycler. Sequences were determined using an ABI377 automated sequencer, and sequence traces were manually analysed for the presence of heterozygous peaks. Base changes were confirmed by sequencing of an independent PCR product.

### Southern analysis

Five μg of genomic DNA from each relevant individual were digested overnight separately with 20U of selected infrequent-cutting restriction enzymes (*Eco*RI, *Kpn*I, *Sma*I, *Sac*I, *Sac*II; New England Biolabs). Samples were electrophoresed through 0.8% agarose gels and transferred to Hybond N nylon membranes (Amersham) using standard protocols (Sambrook et al, 1989). Following transfer, membranes were rinsed in 2XSSC, dried, and UV-crosslinked. For hybridization, filters were incubated at 65°C in 100 ml hybridization solution (7% SDS, 0.263 M $Na_2HPO_4$ pH 7.2, 1 mM EDTA pH 8.0, 1% BSA) for 2–4 hours prior to addition of probe, then hybridized overnight in 10 ml of the same hybridization solution. Two low (2×SSC/0.1% SDS) and two high (0.1×SSC/0.1% SDS) stringency washes were performed at 65°C, and filters were exposed to Fuji autoradiographic film at –70°C for 48 hours. A probe was generated by restriction digestion from a *MEN1* cDNA containing the entire coding region of the gene. Probes were radiolabelled with [α-$^{32}$P]dCTP using a Rediprime II random primer labelling kit (Amersham) as per the manufacturer's instructions.

### RESULTS AND DISCUSSION

### Identification of MEN1 gene mutations

A total of 12 patients from MEN 1 families, 8 sporadic MEN 1 cases, one case of familial acromegaly, 5 unrelated patients with FIHPT, and 7 patients demonstrating MEN 1-like symptoms were analysed in this study for the presence of germline mutations of the *MEN1* gene. This was achieved primarily by sequence analysis of exon-specific PCR products from the coding region of the gene. A summary of the clinical details of the patients analysed is presented in Table 2. Germline mutations were identified in 10

 © 2000 Cancer Research Campaign

**Table 2** Clinical presentations of MEN 1 and MEN 1-like patients with *MEN1* gene mutations identified.

**MEN 1 Families:**

| ID | Clinical features | No. affected | Lesions | Exon | *MEN1* mutation | Effect on protein |
|---|---|---|---|---|---|---|
| 20002 | ZES, ↑PP | 5 | HPT | 10 | 7773insC | Frameshift |
| 30001 | | 4 | Pituitary adenoma, multicentric insulinoma, parathyroid adenoma, HPT | | Partial gene deletion†,* | lack of expression |
| 40933 | | 4 | Prolactinoma, insulinoma, pituitary adenoma, HPT | 2 | 2536del4 | frameshift |
| 41121 | Acromegaly, renal calculi | 4 | HPT | | not found | |
| 41131 | Renal calculi, ZES | 4 | Gastrinoma, HPT, pituitary adenoma, lipomata | 9 | 7361del11‡ | deletes exon/ intron border |
| 41177 | ZES, ↑PP, ↑gastrin, pancreatitis | 3 | Adrenal tumour, HPT | 8 | 6630T>G | Y353D |
| 41178 | | 4 | Gastrinoma, pituitary tumour, HPT | 8 | 6690C>T | P373S |
| 41179 | renal calculi | 4 | Pancreatic tumour, thymic carcinoid, prolactinoma, HPT | 9 | 7312delG≠ | frameshift |
| 41180 | | 2 | Prolactinoma, HPT | 3 | 4482del4 | frameshift |
| 50000 | ↑gastrin, duodenal ulcer, ZES | 4 | Lipomata, islet cell tumour, HPT, gastrinoma, pancreatic adenoma | 10 | 7916AGC> T | frameshift |
| 60004 | renal calculi, dyspepsia, galactorrhoea | 3 | Gastrinoma, prolactinoma, malignant thymoma, HPT | 9 | 7254G>C | R415P |
| 96002 | renal calculi | 4 | Lung and thymic carcinoids, prolactinoma, non-functioning pituitary, insulinoma, HPT | 9 | 7278G>A† | W423X |

**Sporadic MEN 1 patients:**

| ID | Clinical features | No. affected | Lession | Exon | *MEN1* mutation | Effect on protein |
|---|---|---|---|---|---|---|
| 10000 | Acromegaly | 1 | Parathyroid adenoma | | not found | |
| 24000 | ↑prolactin, $Ca^{2+}$; ↓ phosphate | 1 | Insulinoma | | not found | |
| 40931 | Hypercalcaemia, acromegaly | 1 | Acidophilic pituitary adenoma, HPT, lipomata, bowel polyps | | not found | |
| 41076 | Acromegaly | 1 | Prolactinoma, HPT | | not found | |
| 41120 | Galactorrhoea | 1 | Prolactinoma | | not found | |
| 41181 | | 1 | Hyperplastic adrenal, prolactinoma, HPT, insulinoma, glucagonoma | 10 | 7622C>T | R460$^{x}$ |
| 50001 | Cushing's disease | 1 | ACTH pituitary adenoma, HPT | | not found | |
| 60005 | | 1 | Non-functioning pituitary, HPT | | not found | |

**MEN 1-like patients/families**

| ID | Clinical features | No. affected | Lesions | Exon | *MEN1* mutation | Effect on protein |
|---|---|---|---|---|---|---|
| 20005 | Hypoglycemia, acromegaly | 5 | Bronchial carcinoid, caecalsarcoma | | not found | |
| 41025 | | 5 | Pituitary tumour, HPT, craniopharyngiema | | not found | |
| 41082 | | 2 | Insulinoma, MTC | | not found | |
| 41174 | Hypertension, stomach ulcer | 3 | Thyroid adenoma, HPT | 4 | 4747G>T | R229L |
| 41175 | ↑PP, peptic ulcer, acromegaly | 3 | HPT, GH, prolactin-secreting pituitary adenoma | | not found | |
| 41176 | Renal calculi | 1 | Sporadic recurrent HPT | | not found | |
| 70004 | | 2 | Non-secretory pancreatic, HPT | | not found | |

**Familial isolated hyperparathyroidism/acromegaly families:**

| ID | Clinical features | No. affected | Ages at diagnosis | Exon | MEN1 mutation | Effect on protein |
|---|---|---|---|---|---|---|
| 40883 | familial acromegaly | 3 | | | not found | |
| 41067 | FIHPT | 3 | 20–40 | 2 | 2543ins18 | 6 amino acid insertion |
| 41173 | FIHPT | 2 | 29 | | not found | |
| 41182 | FIHPT | 5 | | | not found | |
| 41183 | FIHPT | 2 | 38 | | not found | |
| 70013 | FIHPT | 4 | 18–62 | | not found | |

Abbreviations: HPT = hyperparathyroidism; FIHPT = familial isolated hyperparathyroidism; ZES = Zollinger–Ellison syndrome; PP = pancreatic polypeptide; MTC = medullary thyroid carcinoma. † incorrectly reported as no mutation in Teh et al (1998a). * Further information in Figures 4 and 5. ‡ Incorrectly reported as 7352del11 in Teh et al (1998a). ≠ Incorrectly reported as 4340insA in Teh et al (1998a).

© 2000 Cancer Research Campaign

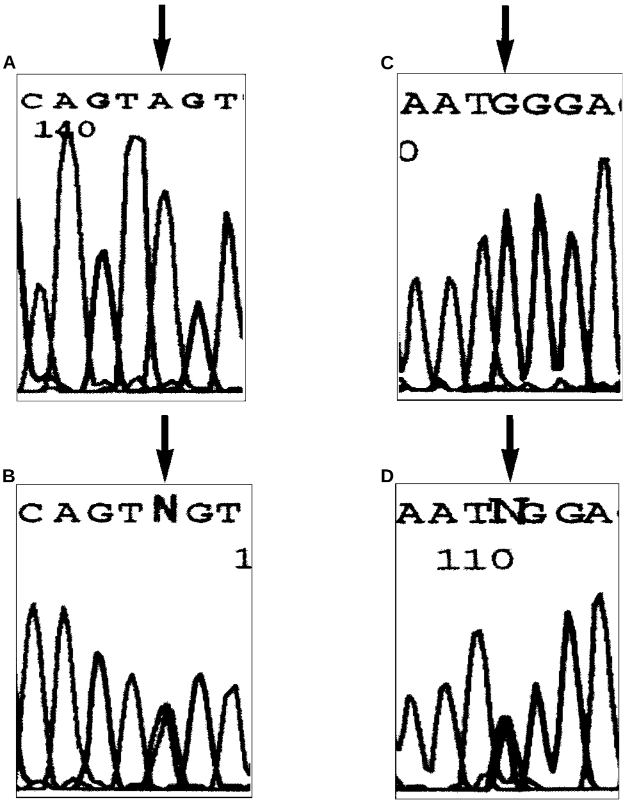

**Figure 1** Sequence traces for: (**A**) a portion of wild type exon 8 (complementary strand); (**B**) A–C transversion on reverse strand responsible for missense mutation Y353D; (**C**) wild type exon 9; (**D**) G–A transition resulting in W423[x]

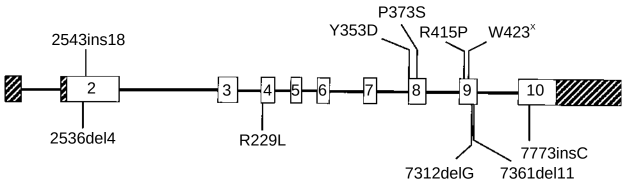

**Figure 2** Schematic diagram of the *MEN1* gene showing germline mutations identified. 2543ins18 was identified in a patient with FIHPT, R229L was found in a patient with MEN 1-like symptoms, and the remaining mutations were identified in MEN 1 patients

MEN 1 families, one sporadic MEN 1 patient, one patient with FIHPT and one MEN 1-related case (Table 2). Examples of sequence traces showing the base changes responsible for 2 mutations are shown in Figure 1. A schematic diagram of the *MEN1* gene, showing the approximate locations of the mutations reported here is shown in Figure 2. Only 3 of these mutations (2536del4, 7773insC; R460[x]) have been reported previously, either in MEN 1 families or in MEN 1-type sporadic endocrine tumors (Agarwal et al, 1997; Bassett et al, 1998).

As has been previously observed, the mutations are distributed throughout the coding region of the gene, although a relatively large proportion of mutations are located in exon 9. This region forms part of a putative site for interaction with JunD (Agarwal et al, 1999), so it is possible that some of the exon 9 mutations reported here may prevent the interaction between menin and JunD.

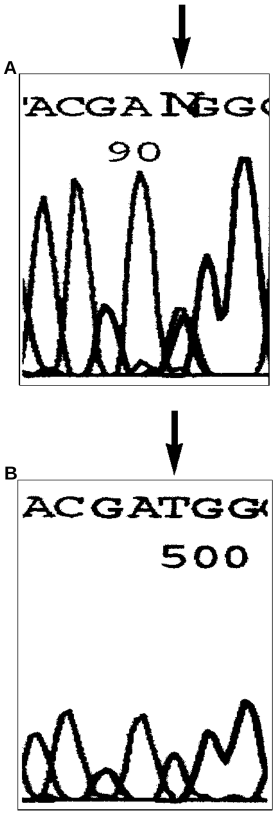

**Figure 3** Sequence traces showing loss of expression of one allele in a MEN 1 patient (ID. 30001). (**A**) A portion of exon 9 sequence from genomic DNA showing a heterozygous D418D polymorphism. (**B**) The same sequence by RT-PCR, showing only expression of the polymorphic allele

There have been several other reports previous to this study of germline mutations identified in patients with FIHPT (e.g. Teh et al, 1998b; Shimizu et al, 1997; Ohye et al, 1998; Fujimori et al, 1998; Poncin et al, 1999), and a number of reports of FIHPT families without *MEN1* gene mutations (Agarwal et al, 1997; Giraud et al, 1998; Teh et al, 1998a). Although mutation of the *MEN1* gene in FIHPT is an uncommon event, it should still be considered in genetic screening protocols.

### Further analysis of patients with no germline mutations

As a lower number of patients were found to carry germline *MEN1* gene mutations than expected, additional analyses were conducted to determine if one copy of the *MEN1* gene was being inactivated by a method not detectable by sequence analysis of genomic DNA. If a heterozygous sequence polymorphism was identified during germline mutation analysis, RT-PCR was conducted (provided LCLs were available for isolation of RNA) to determine whether loss of mRNA expression of one allele was occurring. This was identified in one familial MEN 1 patient (ID: 30001) out of 11 patients analysed, and the sequence traces demonstrating this are shown in Figure 3.

To investigate whether this loss of expression of one allele of the *MEN1* gene was due to some rearrangement or deletion unable to be detected by PCR, genomic DNA from the patient was digested with a series of infrequent-cutting restriction enzymes

*© 2000 Cancer Research Campaign*

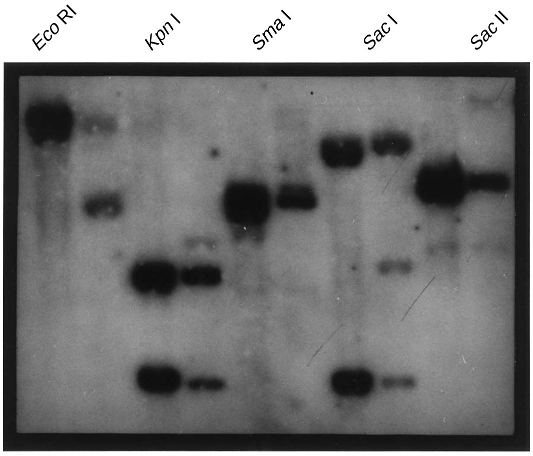

**Figure 4** Southern blot analysis of DNA from a MEN 1 patient carrying a large germline deletion. For each enzyme, lane 1 is from a healthy control, and lane 2 is from the MEN 1 patient with a deletion. So that some of the faint bands can be clearly seen, a long exposure time of the autoradiograph has been deliberately used. Band sizes are detailed in Table 3

**Table 3** Restriction fragment sizes obtained by Southern analysis in Figure 4 of wild type and MEN1 intragenic deletion DNA.

| **Restriction enzyme** | **Fragment sizes (kb)** | |
|---|---|---|
| | **control** | **mutant** |
| *Eco*RI | 12.0 | 12.0 |
| | | 6.5 |
| *Kpn*I | 4.7 | 4.7 |
| | 3.4 | 3.4 |
| | | 5.3 |
| *Sma*I | 6.2 | 6.2 |
| | 4.5 | 4.5 |
| | 5.4 | 1.5 |
| | 1.5 | 12.0 |
| | 1.4 | 15.2 |
| | | 0.9 |
| *Sac*I | 9.2 | 9.2 |
| | 3.4 | 3.4 |
| | 1.1 | 1.1 |
| | | 4.8 |
| *Sac*II | 6.6 | 6.6 |
| | 5.2 | 5.2 |
| | 1.5 | 1.5 |
| | | 15.2 |

and evaluated by Southern analysis. Digestion with some of these enzymes did produce different-sized bands compared to a control sample (Figure 4). In addition, all patients without a germline mutation were analysed in this fashion using the restriction enzymes *Eco*RI and *Kpn*I, and no aberrant-sized bands were observed (data not shown). Twenty known control samples were also screened with *Kpn*I and *Eco*RI to ensure that these aberrant bands were not just due to population variation (data not shown).

The approximate sizes of the bands in Figure 4 were calculated using a standard curve derived from molecular size markers and are shown in Table 3. From analysis of the aberrant bands, it appears that *Sma*I, *Sac*II and *Kpn*I sites known to be located in intron 1 are lost, as is a *Sac*I site in exon 2. An *Eco*RI site expected to lie about 3.5 kb upstream of the *MEN1* gene would be moved

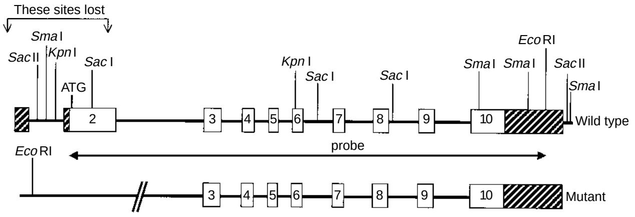

**Figure 5** Schematic diagram of the *MEN1* gene and germline deletion in a MEN 1 family. Restriction sites known to be detected by the probe used are marked to demonstrate the rationale behind the predicted deletion closer as a result of a deletion, thus explaining the smaller bands. From this, it seems that one allele of the *MEN1* gene in this patient contains a large deletion, beginning somewhere upstream of the gene and terminating somewhere before exon 6, thus obliterating the start codon (Figure 5). Analysis with additional restriction enzymes and exon-specific probes may define this deletion further. No unusual clinical features were observed associated with this deletion. Because a similar deletion has been reported previously (Kishi et al, 1998), we suggest such analysis should become a standard procedure in mutation screening protocols.

It remains possible that the *MEN1* gene is inactivated by mechanisms other than those we have described above, such as mutation of intronic or promoter sequences, which may result in reduced transcription or decreased mRNA stability. Mutation of an intron of the *MEN1* gene has been previously reported (Engelbach et al, 1999), resulting in inclusion of 7 bases of intronic sequence into the mRNA and a truncated protein sequence. A number of patients were screened for *MEN1* gene mutations in this study, with no such similar alterations found. However, screening of promoter and intronic sequences, as well as expression analyses should be considered in *MEN1* screening protocols.

### ACKNOWLEDGEMENTS

We are grateful to the National Health and Medical Research Council of Australia and the Queensland Cancer Fund for supporting this work. We wish to thank all patients and their family members for participating in this study.

### REFERENCES

Agarwal SK, Kester MB, Debelenko LV, Heppner C, Emmert-Buck MR, Skarulis MC, Doppman JL, Kim YS, Lubensky IA, Zhuang Z, Green JS, Guru SC, Manickam P, Olufemi S-E, Liotta LA, Chandrasekharappa SC, Collins FS, Spiegel AM, Burns AL and Marx SJ (1997) Germline mutations of the MEN1 gene in familial multiple endocrine neoplasia and related states. *Hum Mol Genet* **6**: 1169–1175

Agarwal SK, Guru SC, Heppner C, Erdos MR, Collins RM, Park SY, Saggar S, Chandrasekharappa SC, Collins FS, Spiegel AM, Marx SJ and Burns AL (1999) Menin interacts with the AP1 transcription factor JunD and represses JunD-activated transcription. *Cell* **96**: 143–152

Bassett JHD, Forbes SA, Pannett AAJ, Lloyd SE, Christie PT, Wooding C, Harding B, Besser GM, Edwards CR, Monson JP, Sampson J, Wass JAH, Wheeler MH and Thakker RV (1998) Characterization of mutations in patients with multiple endocrine neoplasia type 1. *Am J Hum Genet* **62**: 232–244

Chandrasekharappa SC, Guru SC, Manickam P, Olufemi S-E, Collins FS, Emmert-Buck MR, Debelenko LV, Zhuang Z, Lubensky IA, Liotta LA, Crabtree JS, Wang Y, Roe BA, Weisemann J, Boguski MS, Agarwal SK, Kester MB, Kim YS, Heppner C, Dong Q, Spiegel AM, Burns AL and Marx SJ (1997) Positional cloning of the gene for multiple endocrine neoplasia-type 1. *Science* **276**: 404–407

*© 2000 Cancer Research Campaign* 

Darling TN, Skarulis MC, Steinberg SM, Marx SJ, Spiegel AM and Turner M (1997) Multiple facial angiofibromas and collagenomas in patients with multiple endocrine neoplasia type 1. *Arch Dermatol* **133**: 853–857

Engelbach M, Forst T, Hankeln T, Tratzky M, Heerdt S, Pfutzner A, Kann P, Kunt T, Schneider S, Schmidt ER and Beyer J (1999) Germline mutations in the MEN1 gene:creation of a new splice acceptor site and insertion of 7 intron nucleotides into the mRNA. *Int J Mol Med* **4**: 483–485

Fujimori M, Shirahama S, Sakurai A, Hashizume K, Hama Y, Ito K, Shingu K, Kobayashi S, Amano J and Fukushima Y (1998) Novel V184E germline mutation in a Japanese kindred with familial hyperparathyroidism. *Am J Med Genet* **80**: 221–222

Giraud S, Zhang CX, Serova-Sinilnikova O, Wautot V, Salandre J, Buisson N, Waterlot C, Bauters C, Porchet N, Aubert J-P, Emy P, Cadiot G, Delemer B, Chabre O, Niccoli P, Leprat F, Duron F, Emperauger B, Cougard P, Goudet P, Sarfati E, Riou J-P, Guichard S, Rodier M, Meyrier A, Caron P, Vantyghem M-C, Assayag M, Peix J-L, Pugeat M, Rohmer V, Valloton M, Lenior G, Gaudray P, Proye C, Conte-Devolx B, Chanson P, Shugart YY, Goldgar D, Murat A and Calender A (1998) Germ line mutation analysis in patients with multiple endocrine neoplasia type 1 and related disorders. *Am J Hum Genet* **63**: 455–467

Kishi M, Tsukada T, Shimizu S, Futami H, Ho Y, Kanbe M, Obara T and Yamaguchi K (1998) A large germline deletion of the MEN1 gene in a family with multiple endocrine neoplasia type 1. *Japan J Cancer Res* **81**: 1–5

Komminoth P, Heitz PU and Klöppel G (1998) Pathology of MEN 1:morphology, clinicopathologic correlations and tumour development. *J Intern Med* **243**: 455–464

Larsson C, Skogseid B, Öberg K, Nakamura Y and Nordenskjöld M (1988) Multiple endocrine neoplasia type 1 gene maps to chromosome 11 and is lost in insulinoma. *Nature* **332**: 85–87

Lemmens I, Van de Ven WJM, Kas K, Zhang CX, Giraud S, Wautot V, Buisson N, De Witte K, Salandre J, Lenoir G, Pugeat M, Calender A, Parente F, Quincey D, Gaudray P, De Wit MJ, Lips CJM, Höppener JWM, Khodaei S, Grant AL, Weber G, Kytölä S, Teh BT, Farnebo F, Phelan C, Hayward N, Larsson C, Pannett AAJ, Forbes SA, Bassett JHD and Thakker RV (1997) Identification of the multiple endocrine neoplasia type 1 (MEN 1) gene. *Hum Mol Genet* **6**: 1177–1183

Miller SA, Dykes DD and Polesky HF (1988) A simple salting out procedure for extracting DNA from human nucleated cells. *Nucl Acids Res* **16**: 1215

Ohye H, Sato M, Matsubara S, Miyauchi A, Imachi H, Murao K and Takahara J (1998) Germline mutation of the multiple endocrine neoplasia type 1 (MEN 1) gene in a family with primary hyperparathyroidism. *Endocrine J* **45**: 719–723

Poncin J, Abs R, Velkeniers B, Bonduelle M, Abramowicz M, Legros JJ, Verloes A, Meurisse M, Van Gaal L, Verellen C, Koulischer L and Beckers A (1999) Mutation analysis of the MEN1 gene in Belgian patients with multiple endocrine neoplasia type 1 and related diseases. *Hum Mutat* **13**: 54–60

Sambrook J, Fritsch EF and Maniatis T (1989) *Molecular Cloning: A Laboratory Manual, 2nd Ed*. Cold Spring Harbour Laboratory Press, USA

Shimizu S, Tsukada T, Futami H, Ui K, Kameya T, Kawanaka M, Uchiyama S, Aoki A, Yasuda H, Kawano S, Ito Y, Kanbe M, Obara T and Yamaguchi K (1997) Germline mutation of the MEN1 gene in Japanese Kindred with multiple endocrine neoplasia type 1. *Japan J Cancer Res* **88**: 1029–1032

Teh BT, Grimmond S, Shepherd J, Larsson C and Hayward N (1995) Multiple endocrine neoplasia type 1: clinical syndrome to molecular genetics. *Aust NZ J Surg* **65**: 708–713

Teh BT, Kytölä S, Farnebo F, Bergman L, Wong FK, Weber G, Hayward N, Larsson C, Skogseid B, Beckers A, Phelan C, Edwards M, Epstein M, Alford F, Hurley D, Grimmond S, Silins G, Walters M, Stewart C, Cardinal J, Khodaei S, Parente F, Tranebjærg L, Jorde R, Menon J, Khir A, Tan TT, Chan SP, Zaini A, Khalid BAK, Sandelin K, Thompson N, Brandi M-L, Warth M, Stock J, Leisti J, Cameron D, Shepherd JJ, Öberg K, Nordenskjöld M and Salmela P (1998a) Mutation analysis of the MEN1 gene in multiple endocrine neoplasia type 1, familial acromegaly and familial isolated hyperparathyroidism. *J Clin Endocrinol Metab* **83**: 2621–2626

Teh BT, Esapa CT, Houlston R, Grandell U, Farnebo F, Nordenskjöld M, Pearce CJ, Carmichael D, Larsson C and Harris PE (1998b) A family with isolated hyperparathyroidism segregating a missense MEN1 mutation and showing loss of the wild type alleles in the parathyroid tumours. *Am J Hum Genet* **63**: 1554–1549

*© 2000 Cancer Research Campaign*
